# Thyroid Hormone May Regulate mRNA Abundance in Liver by Acting on MicroRNAs

**DOI:** 10.1371/journal.pone.0012136

**Published:** 2010-08-13

**Authors:** Hongyan Dong, Martin Paquette, Andrew Williams, R. Thomas Zoeller, Mike Wade, Carole Yauk

**Affiliations:** 1 Environmental Health Sciences and Research Bureau, Health Canada, Ottawa, Ontario, Canada; 2 Molecular and Cellular Biology Program, University of Massachusetts, Amherst, Massachusetts, United States of America; Institute of Genetics and Molecular and Cellular Biology, France

## Abstract

MicroRNAs (miRNAs) are extensively involved in diverse biological processes. However, very little is known about the role of miRNAs in mediating the action of thyroid hormones (TH). Appropriate TH levels are known to be critically important for development, differentiation and maintenance of metabolic balance in mammals. We induced transient hypothyroidism in juvenile mice by short-term exposure to methimazole and perchlorate from post natal day (PND) 12 to 15. The expression of miRNAs in the liver was analyzed using Taqman Low Density Arrays (containing up to 600 rodent miRNAs). We found the expression of 40 miRNAs was significantly altered in the livers of hypothyroid mice compared to euthyroid controls. Among the miRNAs, miRs-1, 206, 133a and 133b exhibited a massive increase in expression (50- to 500-fold). The regulation of TH on the expression of miRs-1, 206, 133a and 133b was confirmed in various mouse models including: chronic hypothyroid, short-term hyperthyroid and short-term hypothyroid followed by TH supplementation. TH regulation of these miRNAs was also confirmed in mouse hepatocyte AML 12 cells. The expression of precursors of miRs-1, 206, 133a and 133b were examined in AML 12 cells and shown to decrease after TH treatment, only pre-mir-206 and pre-mir-133b reached statistical significance. To identify the targets of these miRNAs, DNA microarrays were used to examine hepatic mRNA levels in the short-term hypothyroid mouse model relative to controls. We found transcripts from 92 known genes were significantly altered in these hypothyroid mice. Web-based target predication software (TargetScan and Microcosm) identified 14 of these transcripts as targets of miRs-1, 206, 133a and 133b. The vast majority of these mRNA targets were significantly down-regulated in hypothyroid mice, corresponding with the up-regulation of miRs-1, 206, 133a and 133b in hypothyroid mouse liver. To further investigate target genes, miR-206 was over-expressed in AML 12 cells. TH treatment of cells over-expressing miR-206 resulted in decreased miR-206 expression, and a significant increase in two predicted target genes, Mup1 and Gpd2. The results suggest that TH regulation of these genes may occur secondarily via miR-206. These studies provide new insight into the role of miRNAs in mediating TH regulation of gene expression.

## Introduction

Thyroid hormones (TH) are critically important for development, tissue differentiation, and maintenance of metabolic balance in mammals through direct and indirect regulation of expression in target genes [1]. Severe disruption of TH action during fetal and early neonatal development leads to a suite of permanent deficits in experimental animals and humans [1]. The liver plays a critical role in metabolism, serum glucose and lipid regulation and is a major target organ of TH. Previous studies using comprehensive transcriptional arrays have shown that TH regulates the expression of genes involved in these important physiological processes [2], [3]. However, the mechanism by which TH regulates the expression of these genes, whether by direct actions on transcriptional activity or by indirect actions on mechanisms that control cellular levels of mRNAs, is not well understood.

MicroRNAs (miRNAs) are small non-coding RNAs of 19–24 nucleotides in length that are important regulators of crucial biological processes, such as metabolism, cell growth, apoptosis and carcinogenesis [4], [5]. The number of known miRNAs has rapidly increased over the past years. Recently, the Sanger Institute released the latest version of their database of known miRNAs (miRBase 14.0; Sep 2009, http://microrna.sanger.ac.uk); 786 mature mouse miRNA sequences are currently reported. Long primary miRNAs are transcribed by RNA polymerase II in the nucleus, and then modified by an enzyme complex containing DROSHA and DGCR8 to form pre-miRNA. Subsequent cleavage of pre-miRNA by an RNase III, DICER 1, results in mature miRNA, which suppresses translation and enhances degradation of target gene transcripts by binding to complementary regions within the target transcripts [4], [5].

Considering the importance of TH in regulating fundamental processes governed by hepatic function, and the potential importance of miRNAs in regulating genes coding for proteins important in these function, we sought to test the hypothesis that TH regulates specific miRNAs. Therefore, we employed DNA microarrays and TaqMan low density arrays (TLDA) to analyze gene and miRNA expression profiles in a juvenile hypothyroid mouse model induced by short-term exposure of dams to goitrogen before weaning. TLDA offers a high throughput and sensitive approach for detection of miRNAs [6]. Selected miRNAs were examined in more detail in other animal models with altered TH levels and in an *in vitro* system to confirm TH regulation. The target genes of one miRNA (miR-206) were investigated with cell lines stably transfected with the corresponding miRNA. Analysis of miRNA expression changes in combination with global mRNA levels provides a powerful approach to determine the effect of TH perturbation on miRNA expression and function. The results of this study provide insight into the role of miRNAs in mediating the actions of TH on liver function.

## Materials and Methods

### Ethics Statement

All animal care and handling was in accordance with Canadian Council for Animal Care Guidelines and was approved by the Health Canada Animal Care Committee. Permit number is 2007006.

### Animal models with altered TH level

C57BL/6 mice were purchased from Charles River (St. Constant, QC, Canada) and were housed in hanging polycarbonate cages under a 12∶12 hour light-dark cycle at 23°C with food (Purina rodent chow 5010; Ralston-Purina, St. Louis, MO, USA) and sugar water (1%) available *ad libitum*. All cages contained shelters and nesting material. After a 10 day acclimation period breeding was initiated by transferring two sexually mature female mice (8 weeks post natal) into the home cage of a sexually mature (10 weeks) male. After 4 nights of co-housing each female was transferred to a separate cage. Dams were allowed to litter naturally and litter numbers were not adjusted. On post natal day (PND) 12, half of the dams with litters were provided with sugar water containing methimazole (MMI, 0.05%, Sigma Chemical, St. Louis, MO) and perchlorate (1%, Sigma chemical), while other half were provided sugar water. Four hours before sacrifice on PND 15, pups with sugar water were injected with PBS (control group) or T4/T3 (hyper group, 50/5ug/100gbw); MMI and perchlorate treated pups were injected with PBS (hypo group) or T4/T3 (hypo+ group, 20/2ug/100gbw).

For PTU induced hypothyroidism, C57BL/6 time-pregnant mice (13-day gestation, GD 13) were purchased from Charles River (St. Constant) and supplied *ad libitum* with 0.3% diet cherry Kool-aid in water containing 0%(control) or 0.04% PTU (6-propylthiouracil; Sigma Chemical) from GD 13 to PND 15.

On PND 15, a male pup from each litter was sacrificed by exsanguination under isofluorane anaesthesia. Serum, prepared using Serum Separator Tubes (BD Biosciences, Mississauga, ON, Canada) was retained for T4 analysis with RIA kits (MP Biomedicals, Medicorp, Montreal, QC, Canada). Liver was dissected as rapidly as possible, immediately frozen in liquid nitrogen and stored at −80°C.

### Cell culture and stable transfection

Mouse hepatocyte cells AML 12 (ATCC, Manassas, VA,USA) were grown at 37°C in Dulbecco's modified Eagle's medium/Ham's F-12 nutrient mixture with 10% fetal Bovine serum, 100 nM dexamethasone and ITS (insulin, transferrin and selenium, Invitrogen, Burlington, ON, Canada). Stable transfection was performed using 18 µl FuGene (Roche, Indianapolis, IN, USA) with 12 µg miRNASelect pEGP-mir Null Control Vector or pEGP-mmu-mir-206 Expression Vector (Cell Biolabs, Inc., San Diego, CA, USA) in 10-mm Petri dishes. Green clones in the medium containing 2 µg/ml puromycin (Invitrogen) were identified by microscopy, and were picked and cultured in completed medium with puromycin for one week. The over expression of miR-206 was confirmed with qRT-PCR. Ten percent stripped fetal bovine serum (Medicorp, QC, Canada) was added to cultured cells one day before cells were incubated with or without 10 nM T3.

### RNA Extraction and Purification

For microarray analysis, total RNA was extracted with TRIzol reagent (Invitrogen) followed by RNeasy Mini Kit (Qiagen, Mississauga, ON, Canada) clean-up according to the manufacturer's instructions. RNA integrity was determined using an Agilent 2100 Bioanalyzer (Agilent Tech. Inc. Mississauga, ON, Canada) and only high quality RNA (RIN>9.0) was used for microarray analysis. For other analyses (miRNA expression, qRT-PCR), total RNA was extracted with *mir*Vana miRNA Isolation Kits (Ambion, Inc., Applied Biosystems, Foster city, CA, USA) according to the manufacturer's instructions.

### Microarray hybridization

Relative transcript levels were determined using a 2 colour reference hybridization design where each RNA sample was labelled with Cyanine 5-CTP (Cy5) and universal mouse reference total RNA (Stratagene, Cedar Creek, TX, USA) was labelled with Cyanine 3-CTP (Cy3) using Agilent Quick Amp Labelling kits (Agilent Tech. Inc.). Labelled sample and common reference cRNA were hybridized to Agilent mouse oligo microarrays (product number G4122F; 4X44K arrays) according to the manufacturer's instructions. Slides were washed and scanned on an Agilent G2505B microarray scanner and data were acquired with Agilent Feature Extraction software version 10.1.1.1.

### Microarray normalization and analysis of differential gene expression

Background fluorescence was measured using the (-)3xSLv1 probes. Probes exhibiting median signal intensities less than the trimmed mean (trim = 5%) plus three trimmed standard deviations of the (-)3xSLv1 probe were flagged as absent (within the background signal). Data were normalized using the transform.madata function in the MAANOVA library in R using a global lowess with a span of 0.2 [7], [8]. Genes that were differentially expressed as a result of treatment were determined using the MAANOVA library in R. The Fs statistic [9] was used to test for treatment effects. P-values were estimated by the permutation method using residual shuffling, followed by adjustment for multiple comparisons using the false discovery rate (FDR) approach [10]. The fold change calculations were based on the least-square means. Significant genes were identified as those where the Fs statistic had a Benjamini-Hochberg corrected p<0.05. All data are MIAME compliant and available through the Gene Expression Omnibus (GEO, accession number GSE21277).

### MiRNA expression analysis with TLDA

Four male mice from each of the control and hypo groups respectively were used to comprehensively analyze miRNA expression. Samples containing 750 ng RNA were used to perform reverse transcription with the Taqman miRNA Reverse Transcription kit and Megaplex RT Primers Rodent Pool A and B (Applied Biosystem). RT-PCR reactions were performed with TaqMan Rodent miRNA Array A and B (containing up to 600 rodent miRNAs) by the Institut de Recherche en Immunologie et en Cancérologie (IRIC), University de Montreal, with the 7900 HT system. Using the log2 of the delta Ct values, differentially expressed miRNAs were identified using an F-test with U6 as a housekeeping miRNA. The critical value of the F-test statistic was determined by bootstrapping the residuals from the one way ANOVA model [11] using the R [7] software. Residuals were re-sampled within each treatment condition to avoid making the common variance assumption [12]. Multiple comparison adjustment was applied to the final results using the FDR approach [10]. The dataset is available through GEO (accession number GSE21277).

### miRs-1, 206, 133a and 133b expression in animal models or cultured cell lines

Taqman miRNA Reverse Transcription kits (Applied Biosystem) were used for reverse transcription reactions with 10 ng total RNA as template and specific primers from the Taqman miRNA Assay Kits. PCRs were performed with Taqman universal PCR Master Mix according to the manufacturer's instructions. Three animals from each group or 3 batches of cultured cells were used. Relative miRNA expression was analyzed using the ΔΔCt method with U6 as a housekeeping miRNA and one of the control samples as the calibrator. Significant differences in expression were determined using a Student's t-test and called significant if p<0.05.

### MiRNA target predictions

TargetScan mouse 5.1 (http://www.targetscan.org/mmu_50/) and MicroCosm Targets Version 5 (http://www.ebi.ac.uk/enright-srv/microcosm/cgi-bin/targets/v5/search.pl) were used to predict the targets of miRs-1, 206, 133a and 133b. Genes predicted by either of algorithm were considered to be the targets. These softwares apply different algorithms to identify the highly complementary sites and are widely used for miRNA target prediction [13]–[15].

### qRT-PCR analysis of target genes and precursors of miRNAs

Reverse transcription was carried out with SuperScript III (Invitrogen) using SYBR-Green and a GFX system (BioRad, Mississauga, ON, Canada). Primers were designed using Beacon design 2.0 (Premier BioSoft International, Palo Alto, CA, USA). PCR reactions were performed in duplicate, and the values of the threshold cycles were averaged. Gene expression levels were normalized to *Hprt*. PCR efficiency was examined using the standard curve for each gene. The primer specificity was assured by the melting curve for each gene. A Student's t-test was used for statistical evaluation. The sequences of primers are available upon request.

## Results

### Validation of the short-term MMI/perchlorate-induced hypothyroid juvenile mouse model

**Figure 1:**
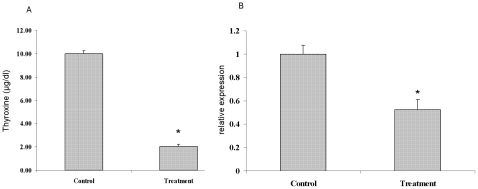
Serum T4 levels and liver malic enzyme mRNA levels in mouse pups rendered hypothyroid by 3 day exposure of dams and their litters to drinking water containing MMI and perchlorate from PND 12 to 15. A. Serum T4 data are expressed as mean ± standard error (SE, n = 10) and * indicates significant difference (p<0.001). B. qRT-PCR for malic enzyme mRNA (positively regulated by direct TH action) was performed with RNA derived from male pups. Data are presented as mean ± SE (n = 5). A two-tailed Student's t-test was used to calculate significance and * indicates p = 0.03.

Serum T4 levels in PND 15 male pups of dams treated from PND 12 to PND 15 with drinking water containing 0.05% MMI/1% perchlorate were significantly reduced (p<0.001, Fig. 1A). Functional hypothyroidism was further confirmed by the observation of a 50% reduction in malice enzyme (a known TH regulated gene in liver [3]) expression in hypothyroid mouse liver (Fig. 1B).

### MiRNA expression in hypothyroid mice

The TLDA analysis revealed that 40 miRNAs were significantly altered (p<0.1) in the liver of hypothyroid mice compared with controls. Among them, 11 miRNAs exhibited a fold change greater than 4 (Table 1); 8 of these 11 (70%) were up-regulated in hypothyroid mice. Three miRNA families (miRs-1 and 206, miRs-133a and 133b as well as miRs-135a and 135b) exhibited very large increases in expression (ranging from 50- to 500-fold). Data for these findings are available through GEO, accession number GSE21277.

**Table 1:** Differentially expressed miRNAs in hypothyroid mouse liver (p<0.1, Fold change >4).

Gene Name	Fold Change	p-value	Clustered miRNA*	Function
**miR-133 family**				
mmu-miR-133b	501.39	0.05	mmu-miR-206	Apoptosis [24], muscle development [25], [26]
mmu-miR-133a	100.36	0.02	mmu-miR-1	Apoptosis [24], muscle development [25], [26]
**miR-1/206 family**				
mmu-miR-1	92.11	0.01	mmu-miR-133a	Apoptosis [24], muscle development [25], [26]
mmu-miR-206	58.90	0.07	mmu-miR-133b	Apoptosis [24], muscle development [25], [26]
**miR-135 family**				
mmu-miR-135b	85.11	0.09		Colorectal cancer [27]
mmu-miR-135a	14.62	0.09		Colorectal cancer [27]
**Others**				
mmu-miR-138*	27.77	0.08		Squamous cell carcinoma [28]
mmu-miR-199b*	4.96	0.05		Choriocarcinoma [29]
mmu-miR-148a*	−4.20	0.04		DNA methyltransferase [30]
mmu-miR-582-5p	−5.14	0.07		Malignant mesothelioma [31]
mmu-miR-200a*	−5.49	0.10		Cervical cancer [32]

*indicates they are in the same chromosome and apart less than 10kb (based on database of TargetScan).

### The expression of miRs-1, 206, 133a, 133b in other animal models with altered TH levels

**Figure 2:**
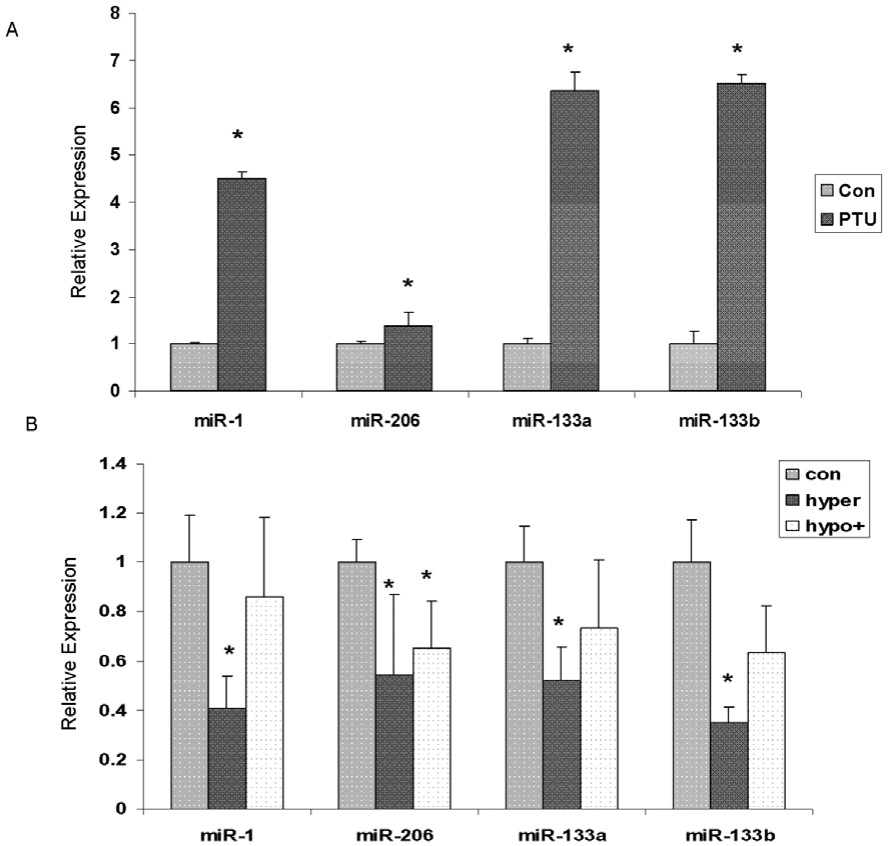
Expression of miRs-1, 206, 133a and 133b in other animal models with altered TH levels. A. Hepatic miRNA expression in livers of PND 15 mouse pups rendered hypothyroid by treatment with drinking water containing 0.04% (wt/vol) of PTU from GD 13 to PND 15. RT-PCR was performed with the Taqman miRNA Assay with RNA derived from male pups (3 per group). B. Hepatic miRNA expression in livers of PND 15 mice whose TH levels were modulated as follows: hyperthyroid pups (hyper) received a *s.c.* injection of T4+T3 (50 µg+5 µg, respectively per 100g bw) four hours prior to sacrifice and corrected hypothyroid pups received drinking water containing MMI and perchlorate (0.05 and 1% wt/vol, respectively) from PND 12 to 15 and an injection of T4+T3 (20 µg+2 µg, respectively per 100g bw) four hours prior to sacrifice, while control mice received an injection of PBS only. RT-PCR was performed with the Taqman miRNA Assay with RNA derived from male pups (3 per group). Data are presented as mean ± SE (n = 3). A two-tailed Student's t-test was used to calculate significance. * indicates p<0.05.

To further investigate the effect of TH on hepatic miRNA expression, we examined the expression of the most differentially regulated miRNAs (miRs-1, 206, 133a, 133b) in the livers of (a) hypothyroid mice induced by PTU treatment (PTU hypothyroid); (b) hyperthyroid mice created by injecting T3/T4 four hours before sacrifice (hyperthyroid) and (c) hypothyroid mice induced by MMI/Perchlorate treatment but receiving T4/T3 injection four hours before sacrifice (corrected hypothyroid). Three mice were chosen from each group and their serum T4 levels were shown in Table 2. As shown in Fig. 2A, all of four selected miRNAs were significantly increased in the livers of PTU induced hypothyroid mice, while significantly decreased in the livers of hyperthyroid mice. Corrected hypothyroid animals had serum T4 levels intermediate between control and hyper thyroid animals although these were only significantly different from the hyperthyroid T4 levels (p = 0.046 vs hyperthyroid and 0.067 vs control; Table 2). Similarly, hepatic expression of all 4 miRNAs was also intermediate between control and hyperthyroid mice with only miR206 being significantly reduced relative to control animals (Fig 2B).

**Table 2:** Serum T4 levels of male pups in the various animal models (n = 3).

	T4 (µg/dl)	p
*Chronical Hypothyroid*		
Control	9.93	
PTU (0.04%)	0.57	0.000
*Transient Models*		
Control	9.6	
Hyper	39.4	0.000
Hypo+	16.6	0.067 (to Control)
		0.046 (to Hyper)

### The expression of miRs-1, 206, 133a and 133b in AML 12 cells treated with TH

**Figure 3:**
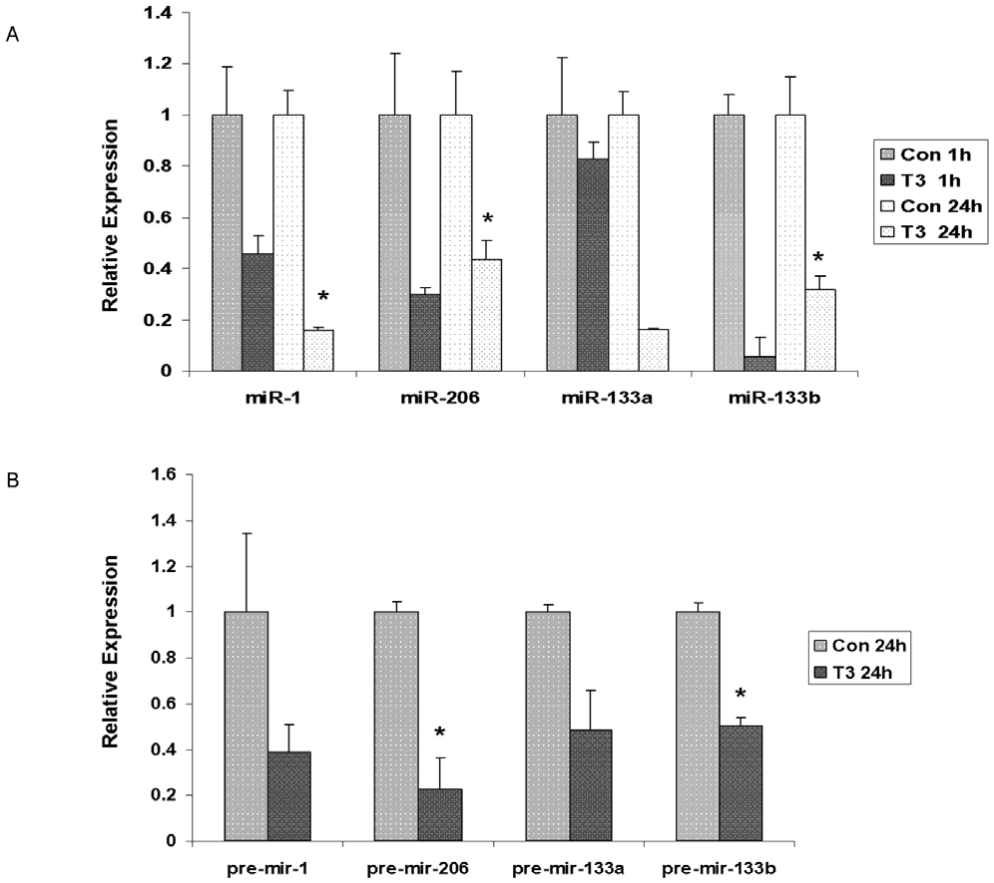
Expression of miRs-1, 206, 133a and 133b as well as their precursors in the AML 12 cells. A. AML 12 cells were treated with 10 nm T3 for 1 hour or 24 hours. The expression of miRs-1, 206, 133a and 133b was examined with the Taqman miRNA Assay. U6 was used as an internal control. Five batches of cultured cells were considered as 5 biological replicates. B. The expression of the precursors of miRs-1, 206, 133a and 133b was examined in AML 12 cell treated with 10 nm T3 for 24 hours with RT-PCR. *Hprt* was used as internal control. Three batches of cultured cells were used as 3 biological replicates. Data are presented as mean ± SE. A two-tailed Student's t-test was used to calculate significance. * indicates p<0.05.

To further explore the effects of TH on miRNA regulation in liver, we treated AML 12 cells (derived from mouse hepatocytes) with 10 nM T3 for 1 hour or 24 hours. The expression of miRs-1, 206, 133a and 133b was examined with the Taqman miRNA Assay. TH treatment caused down-regulation of all miRNAs examined at both 1 hour and 24 hours, although this effect was only statistically significant for miRs-1, 206 and 133b at 24 hours (Fig. 3A).

Since mature miRNAs are derived from the cleavage of precursors by the RNase-III enzyme DICER, we investigated the effects of TH on the levels of the precursors of the selected miRNAs in AML 12 cells. As significant decreases of mature miRNAs were only found at 24 hours, we examined the precursor miRNAs at 24 hours as well. Precursors of all 4 miRNA species were reduced by at least 50% and this reduction was statistically significant (p<0.05) for mir-206 and mir-133b (Fig. 3B) even with the small sample size used.

### Target Genes of miRs-1, 206, 133a and 133b in hypothyroid mouse liver

The livers of five male pups from the transient hypothyroid model were analysed using Agilent gene expression microarrays alongside controls. Significantly altered genes (p<0.05) were found for 103 transcripts between euthyroid and hypothyroid pups. Among them, 92 genes have known functions and include genes known to be regulated by TH, such as Dio1, Spot 14 and Vldlr [3]. The expression of malic enzyme was also decreased in these hypothyroid mice (1.4-fold, unadjusted p-value = 0.005), consistent with the result of qRT-PCR (Fig. 1B). Detailed analysis of the expression profiles of these mice will be presented as part of another publication (Paquette *et al.*, in preparation). The dataset is available through GEO (accession number GSE21277). Because miRs-1and 206 as well as miRs-133a and 133b families exhibited such large fold changes, the targets of these miRNAs were predicted using TargetScan and MicroCosm. The overlap between predicted target genes and significantly changed genes with fold change >1.5 and p<0.05 in hypothyroid mice is shown in Table 3. Of the 14 predicted targets, 11 were significantly down-regulation in hypothyroid pups, corresponding with the up-regulation of miRs-1, 206, 133a and 133b. None of the targets of miRs-1, 206, 133a and 133b that were curated in TarBase (http://diana.cslab.ece.ntua.gr/tarbase/) were altered in the hypothyroid mouse livers.

**Table 3:** Targets of miRs-1, 206, 133a and 133bthat were significantly altered in hypothyroid mouse liver (p<0.05, Fold change >1.5).

Accession Number	Gene Symbol	p-value	Fold Change	Description
**Targets of miR-1/206**				
NM_013703^a^	Vldlr*	0.001	1.669	very low density lipoprotein receptor
NM_001013785^a^	Akr1c19	0.000	1.612	aldo-keto reductase family 1, member C19
NM_029692^a^	Upp2	0.008	−1.562	uridine phosphorylase 2
NM_010274^b^	Gpd2	0.000	−1.695	glycerol phosphate dehydrogenase 2, mitochondrial
NM_031188^a^	Mup1	0.003	−1.815	major urinary protein 1
NM_008737^b^	Nrp1*	0.000	−2.000	neuropilin 1
NM_026460^a^	Serpini2	0.001	−3.080	serine (or cysteine) peptidase inhibitor, clade I, member 2
**Targets of miR-133a/b**				
NM_016919^a,b^	Col5a3	0.000	1.752	procollagen, type V, alpha 3
NM_182959^a^	Slc17a8	0.000	−1.637	solute carrier family 17
NM_025989^a^	Gp2	0.002	−1.727	zymogen granule membrane glycoprotein 2
NM_009344^a^	Phlda1*	0.000	−1.815	pleckstrin homology-like domain, family A, member 1
NM_008693^a^	Klk1b3	0.002	−2.103	kallikrein 1-related peptidase b3
NM_010639^a^	Klk1	0.001	−2.111	kallikrein 1
NM_007769^a^	Dmbt1	0.004	−4.236	deleted in malignant brain tumors 1

*indicates the genes that were found in chronic hypothyroidism in our previous study [3].

a indicates the genes were predicted with the database of MicroCosm Targets and b indicates with TargetScan Mouse.

**Figure 4:**
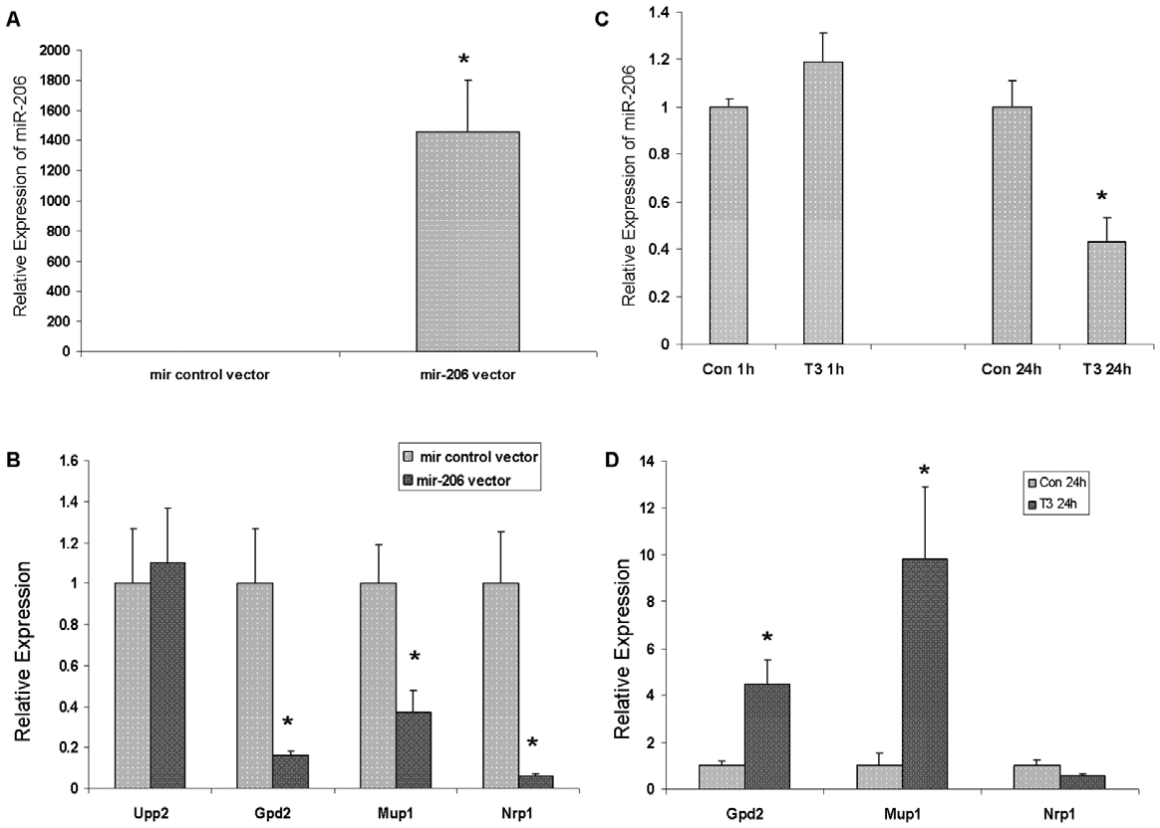
Identification of the target genes of miR-206 that are regulated by TH. A. Levels of miR-206 in AML 12 cells stably transfected to ectopically express miR-206. AML12 cells were transfected with pEGP-mmu-mir-206 expression vector or pEGP-mir null control vector and selected with puromycin and green florescence protein by microscopy. The expression of miR-206 was examined with the Taqman miRNA Assay (n = 5). B. The expression of genes that are putative targets of miR-206 in the two cell types shown in figure 4A was examined with RT-PCR (n = 3). C. The effects of TH on the expression of miR-206 in the transfected cells. The miR-206 transfected cells were treated with 10 nm T3 for 24 hours. MiR-206 expression was analyzed with Taqman miRNA Assay (n = 3). D. The effect of TH on the expression of the miR-206 target genes in the transfected cells. The miR-206 transfected cells were treated with 10 nm T3 for 24 hours. The expression of target genes was examined with RT-PCR (n = 3). Data are presented as mean ± SE. A two-tailed Student's t-test was used to calculate significance. * indicates p<0.05.

To provide direct evidence of the regulation of the expression of target genes by miRNAs in response to TH, we established AML 12 cells that over-expressed mir-206 by stable transfection of vector containing mir-206 precursor. The expression of miR-206 was approximately 1500 times higher in miR-206 over-expressing cells than in mir null control vector transfected cells (Fig. 4A). We selected 4 down-regulated genes from the predicted miR-206 targets in Table 3. The expression of these genes was examined in these cells and the results are shown in Figure 4B. Expression of three out of the four putative target genes were significantly decreased in miR-206 over expressed cells suggesting that these three genes are true targets of miR-206.

To investigate whether these 3 true target genes are regulated by TH, we examined the expression of these 3 genes and that of miR-206 in miR-206 transfected AML 12 cells treated with T3 for 24 hours. T3 treatment decreased the expression of miR-206 by roughly 50% (p<0.05) in miR-206 transfected AML 12 cells (Fig. 4C), while the expression of target genes Gpd2 and Mup1, but not Nrp1, significantly increased (Fig. 4D).

## Discussion

TH are essential for normal development and for normal adult physiology. In both development and in adulthood, an important role of TH is to control metabolism and, perhaps, body weight. Recent studies on the effects of a TR_β_-selective drug that targets the liver indicate that activation of the TR_β_ receptor can lower serum lipids, can decrease global fat stores, and can improve insulin tolerance in *ob/ob* mice [16]. Thus, liver is an important target of TH in regulating energetic metabolism and physiology [2], [3]. TH primarily exert their effects through interaction with TR, which, upon heterodimerization with the retinoic X receptor, acts as ligand–activated transcription factors to initiate or block target gene expression by binding to TH response elements (TREs) in the gene promoter regions. Indeed, much of the focus of work in our laboratory has been on the identification of TREs associated with promoters [17]. However, a direct TR-TRE mechanism may not explain all TH actions. For example, non-genomic action of TH, which is often related to activation of signalling pathways, is another well characterized TH response [18]. MiRNAs represent an additional mechanism by which TRs may regulate or coordinate TH response genes. MiRNAs are involved in many biological processes and functions in controlling protein expression through targeting degradation of mRNA, translational suppression of protein production, and directing chromatin structure (reviewed in [19]). As such, in the current work we explored the role of miRNAs in governing TH response.

To examine the possibility that TH action may influence miRNA expression which could, in turn, alter mRNA levels, we generated hypothyroid mice by short-term treatment with MMI and perchlorate. Global expression of miRNAs and mRNAs were studied with TLDA and DNA microarray technologies. TLDA (a modified qRT-PCR method) was applied to generate miRNA profiles. This high throughput method has increased sensitivity and accuracy relative to microarrays, and demonstrates a 100% miRNA expression validation rate against standard qRT-PCR [6]. We detected 40 significantly altered miRNAs (p<0.1) in the livers of hypothyroid mice. Among these, 11 miRNAs exhibited fold change >4 (Table 1) and included 8 up-regulated and 3 down-regulated miRNAs. Remarkably, miRs-1, 206, 133a and 133b exhibited fold changes in excess of 50-fold. MiR-1 and miR-133a cluster on chromosome 2 within 10 kb, while miR-206 and miR-133b cluster on chromosome 1 within 4 kb. The results demonstrate a highly robust induction of miRNAs in response to hypothyroidism for specific genomic sites.

To demonstrate that the increased miRNA expression was induced by TH deficiency, not the toxicity of MMI and perchlorate, we examined the expression of miRs-1, 206, 133a and 133b in chronic hypothyroid, short-term hyperthyroid and a TH-supplemented transient hypothyroid animal models using RT-PCR. All of the miRNAs significantly increased in the chronically hypothyroid mouse liver, although the fold change was smaller than in the transient hypothyroid model (Fig. 2A). This may be attributed to the different animal models or different detection systems used. Alternatively, there may be some adaptation following chronic hypothyroidism, or the miRNAs may exhibit a large initial response to the TH perturbation. This increased miRNA expression was not seen in TH corrected transient hypothyroidism, while miR-206 was significantly decreased (Fig. 2B). In addition, all selected miRNAs were significantly decreased in hyperthyroid mouse livers. That both serum T4 (Table 2) and hepatic expression of these miRNAs in the corrected hypothyroid animals are intermediate between control and hyperthyroid animal suggests an inverted dose dependant regulation by TH of these miRNA levels. These observations were confirmed in an *in vitro* model of mouse hepatocytes (AML 12 cells), where treatment with T3 caused a rapid (1 hour) reduction in the levels of miRs-1, 206, 133a and 133b. Further reduction of miRs-1 and 133a was found 24 hours post-treatment (Fig. 3A). The expression of miRNA precursors decreased with T3 treatment at 24 hrs (Fig. 3B). Although some of these changes were not significant at the p<0.05 level (only three biological replicates were used in these analyses), the data *in vitro* provide more evidence that TH reduce the cellular levels of miRs-1, 206, 133a and 133b at the transcript level.

TH regulation of these miRNAs is also supported by recent studies on muscle miRNA expression in hypothyroid humans [20]. In this work Visser *et al.* collected skeletal muscle biopsies from clinically hypothyroid patients who were being treated with TH or not. The authors found that TH induced a large down-regulation of primary miRs-206 and 133b. Levels of the primary transcript associated with miRs-1 and 133a were also reduced but to a lesser extent. Together these findings provide strong evidence that TH down-regulate the expression of miRs-1, 206, 133a and 133b in liver and skeletal muscle. However, the targets of these miRNAs and the potential biological implications are not known.

In order to explore the correlation between miRNA and mRNA levels, gene expression microarrays were used to quantify transcript abundance in the livers of the same mice (hypothyroid and euthyroid). Significant alterations in mRNA levels (p<0.05) were found for 92 transcripts with known functions between euthyroid and hypothyroid pups. Changes in gene expression may be regulated by TH through TR-TRE, miRNAs, non-genomic signalling or other mechanisms. Targets of miRs-1, 206, 133a and 133b were predicted using TargetScan and MicroCosm. The analysis predicts that these miRNAs target 14 genes that also exhibited changes in mRNA levels in hypothyroid mice relative to controls (Table 3). The vast majority of these mRNA targets (11/14) were down-regulated under hypothyroid conditions, corresponding with the increased expression of miRs-1, 206, 133a and 133b in these mice. Three targets (Vldlr, Nrp1 and Phlda1) have previously been found to be differentially expressed following alterations in TH levels in mouse livers [3]. We further validated the targets of miR-206 by establishing over-expressing miR-206 in AML 12 cells by stable transfection. The expression of miR-206 was approximately 1500 times higher in the transfected cells relative to control cells (Fig. 4A). The expression of four predicted targets of miR-206 that were down-regulated in hypothyroid livers was examined; 3 of them were decreased significantly in miR-206 transfected cells (Fig. 4B). These findings strongly suggest that these 3 genes are putative targets of miR-206. To further demonstrate that miR-206 targets are regulated by TH, we treated the miR-206 transfected cells with TH for 24 hours and found that the expression of miR-206 decreased significantly (Fig. 4C), and was accompanied by increases in the expression of 2 of the miR-206 target genes (Mup1 and Gpd2, Fig. 4D). Mup1 mediates chemical signalling by acting as pheromone ligand and regulates systemic glucose and lipid metabolism [21], [22], while Gpd2 plays a role in thermogenesis [23]. TH are involved in the regulation of the activities of Mup1 and Gpd2. Our current findings provide possible mechanisms by which TH regulates these activities via post-transcriptional control by miR-206.

These findings suggest that TH may regulate the cellular levels of several species of miRNA which, in turn, regulate the mRNA levels of some genes. To our knowledge, this represents the first observation of miRNA mediating the physiological action of a hormone in the development of the liver. At present, it is not clear if TH reduces the levels of these specific miRNAs by blocking transcription of primary genes or through some other mechanisms. Further confirmation of the target genes and the physiological significance of miRNAs in regulating TH function are underway in our laboratory.

In conclusion, the present study applied global miRNA and mRNA expression profiling to reveal a potential regulatory role for miRNAs in response to TH in the developing mouse liver. Two target genes of miR-206 affected by TH were confirmed *in vitro*. The work provides insight into the mechanisms leading to abnormal metabolism and development in the liver following TH imbalance.
